# Integrating cure and care in Alzheimer’s disease: a systems perspective for precision medicine

**DOI:** 10.3389/fmmed.2026.1960763

**Published:** 2026-10-14

**Authors:** James Arthur Rice, Jean-Marie C. Bouteiller, Ara S. Khachaturian

**Affiliations:** 1 International Neurodegenerative Disorders Research Center (INDRC.cz), Prague, Czechia; 2 Akadimi Foundation, Minneapolis, MN, United States; 3 Arthur J. Gallagher & Co., Rolling Meadows, IL, United States; 4 Institute for Technology and Medical Systems, Alfred E. Mann Department of Biomedical Engineering, Viterbi School of Engineering & Keck School of Medicine, University of Southern California, Los Angeles, CA, United States; 5 Center for Artificial Intelligence and Quantum Computing in System Brain Research, International Neurodegenerative Disorders Research Center, Praha, Czechia; 6 Brain Watch Coalition, Las Vegas, NV, United States; 7 Campaign to Prevent Alzheimer’s Disease, Potomac, MD, United States

**Keywords:** Alzheimer’s disease, artificial intelligence, caregiving, FAIR data, federated learning, microbiome, multi-omics, neuroinflammation

## Abstract

Alzheimer’s disease remains a major global health challenge, with limited disease-modifying therapies and a rapidly growing societal burden. Although amyloid and tau remain central to the field, current evidence also implicates neuroinflammation, immune dysregulation, vascular dysfunction, and host-microbe interactions in disease pathogenesis. These developments support a broader systems framework, but simply combining additional disciplines or data modalities does not by itself preserve the dynamic biology or lived experience of disease. Artificial intelligence multi-omics, and digital biomarkers can help identify clinically meaningful patterns only when data and models preserve relevant structure across time, scale, context, and uncertainty. Realizing this potential will require responsible international data sharing, interoperable infrastructure, explicit evidence provenance, and ethical governance grounded in FAIR principles, context-appropriate explainability, and privacy-preserving approaches such as federated learning. Scientific progress should therefore be measured not only by disease modification, but also by patterns of cognition, behavior, function, quality of life, and caregiver burden. This Perspective argues that cure and care are not competing or merely parallel priorities; they are linked outputs of the same continuously learning research and care ecosystem.

## Graphical Abstract

**Figure:**
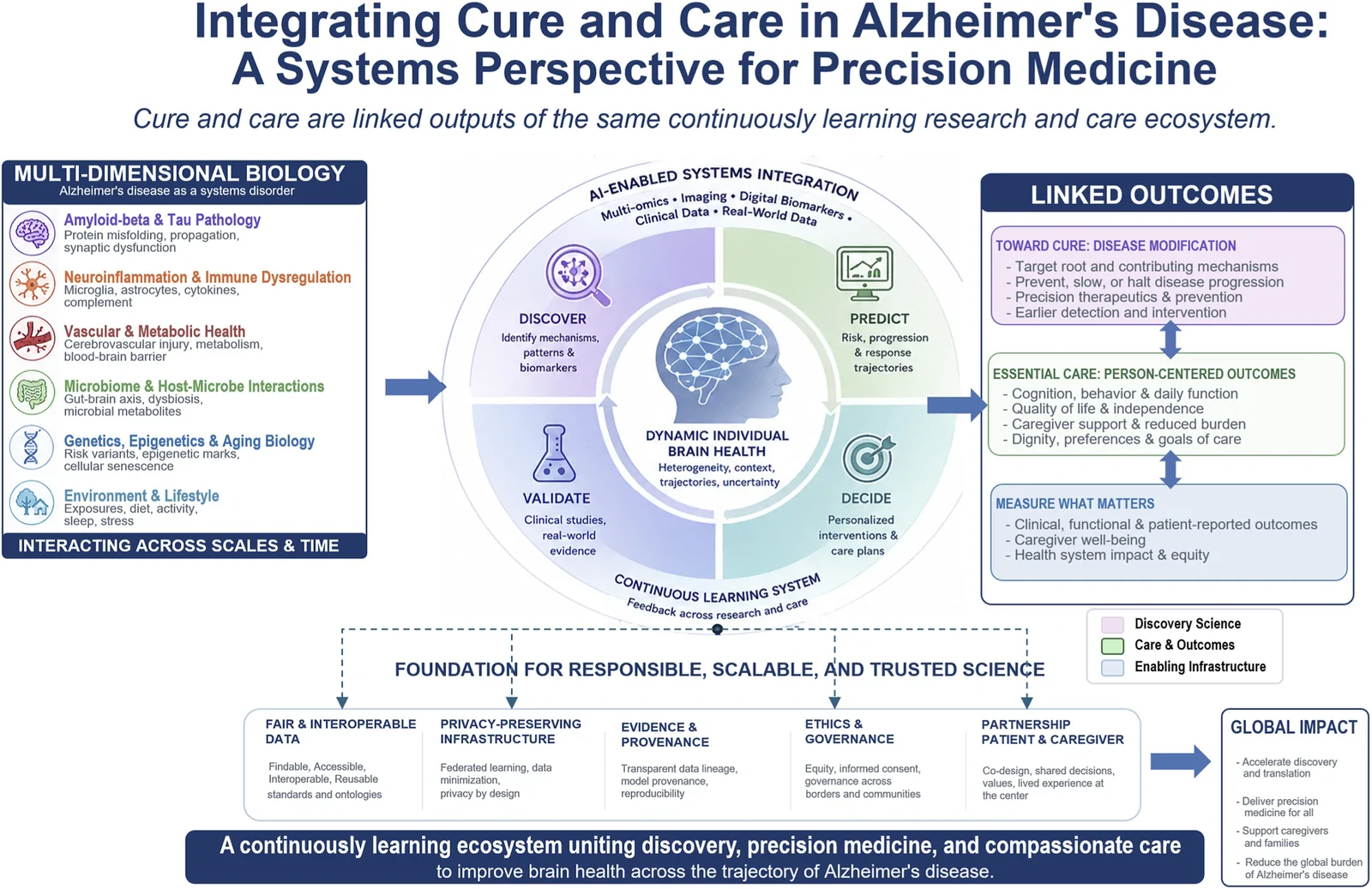

## 1 Introduction

Alzheimer’s disease is increasingly recognized as a complex biological syndrome rather than a disorder driven by a single pathway. The Alzheimer’s disease research landscape is entering an important period of convergence. Advances in molecular biology, microbiology, biomarkers, genetics, neuroimmunology, imaging, and disease-modifying therapeutics are increasingly being connected to earlier detection and clinical intervention. At the same time, the field has recognized a persistent translational gap between scientific discovery, clinical research, and the delivery of evidence-based care (Gregory et al., 2022).

In addition to amyloid-β and tau pathology, the disease is shaped by inflammation, vascular injury, aging biology, metabolic dysfunction, and host-microbe interactions (Livingston et al., 2024; Kakkar et al., 2025). This broader view has important implications for research strategy. If multiple biological systems contribute to disease onset and progression, the challenge is not simply to assemble more data, but to represent interactions across molecular biology, microbiology, neuroscience, and health data over time in ways that support explanation, prediction, and action; such as what Khachaturian and others refer to as building a “brain watch” that enables crafting more accurate and high-precision personalized plans for optimal brain performance and sustained functionality (Khachaturian, 2025; Khachaturian, 2026).

The global burden of dementia continues to rise, and the needs of people living with Alzheimer’s disease cannot wait for a future cure. Caregiver strain, loss of independence, and declining quality of life are immediate realities for millions of families (Alzheimer’s Association, 2024). For this reason, the field should not treat cure and care as parallel workstreams. It should build a research architecture in which biological discovery, clinical implementation, and patient-centered outcomes continuously inform one another.

## 2 Alzheimer’s disease as a systems disorder

A systems perspective is more than a longer list of candidate mechanisms (Morgan et al., 2022). It asks how amyloid-β, tau, immune regulation, vascular health, metabolism, aging biology, and environmental exposures interact across scales and over time to produce heterogeneous patterns of vulnerability, compensation, and clinical expression (Jennifer et al., 2023; Khachaturian et al., 2023; Khachaturian and Khachaturian, 2023). The scientific task is therefore not only to measure each component, but also to represent their relationships without collapsing dynamic processes into static biomarkers or diagnostic categories. This framing supports more precise, individualized approaches to research and care.

A growing body of evidence supports the view that neuroinflammation is not merely a secondary response in Alzheimer’s disease, but part of the disease process itself. Activated microglia, astrocytes, and inflammatory cytokines influence both neuronal injury and repair, depending on timing, intensity, and context (Kakkar et al., 2025). In the short term, immune activation may help clear debris and maintain tissue homeostasis. Over time, however, chronic or dysregulated inflammation can contribute to synaptic dysfunction, neuronal stress, and cell loss.

This immune-centered perspective also helps explain heterogeneity in clinical presentation and progression. Genetic variation in immune-related pathways may influence how individuals respond to pathology, potentially affecting resilience, vulnerability, and treatment response. As a result, immune regulation should be studied not only as a biomarker of disease, but also as a possible therapeutic target.

Microbiology further expands this framework. Interest in the gut-brain-microbiome axis has increased because microbial communities may influence brain health through immune signaling, metabolite production, barrier integrity, and inflammatory regulation (Oso et al., 2026). Because causality remains unproven, microbiome signals should be treated as candidate components of a systems model, with explicit attention to temporality, confounding, and external validation.

## 3 Artificial intelligence and multi-omics integration

The growing complexity of Alzheimer’s biology has created a need for analytic tools that can integrate diverse datasets. The central question, however, is not whether AI can combine more variables, but whether its representations remain faithful to biological and clinical patterns. AI can detect nonlinear relationships across genomics, transcriptomics, proteomics, metabolomics, neuroimaging, electronic health records, and digital biomarkers (Alshehri, 2025), but those relationships remain hypotheses until they are biologically interpreted and externally validated.

The ability of AI to discover meaningful relationships across these diverse data sources depends on more than computational power alone. Robust analyses require standardized data formats, common ontologies, high-quality annotation, and interoperable platforms that enable information from multiple institutions and disciplines to be combined in a consistent manner. Equally important are governance frameworks that support secure, ethical, and privacy-preserving data access. Without these foundations, even the most sophisticated AI models are limited by fragmented, incomplete, or incompatible datasets. Advances in AI must therefore be accompanied by equivalent advances in data stewardship, evidence provenance, missing-data characterization, and collaborative research infrastructure.

In this context, AI should be treated as an inferential instrument rather than a substitute for biological reasoning. Its value lies in identifying candidate patterns, stratifying disease subtypes, predicting patterns, and supporting precision medicine approaches. AI may also help connect molecular signatures with clinically meaningful outcomes such as cognitive decline, functional loss, and treatment response.

Explainability is particularly important. If AI systems are to influence diagnosis, prognosis, or care planning, clinicians must be able to understand the basis of model predictions. The relevant standard is not generic interpretability, but whether the rationale, uncertainty, and limits are intelligible for the specific decision and user. Models that meet this standard are more likely to earn scientific credibility, regulatory acceptance, and patient trust (Shankar et al., 2025). In Alzheimer’s disease, where decisions have profound consequences for patients and families, explainability is not optional.

## 4 Responsible data sharing and representation as scientific infrastructure

AI and multi-omics approaches depend on access to large, diverse, and high-quality datasets. For Alzheimer’s research, responsible data sharing should therefore be treated as scientific infrastructure, not simply as an administrative issue. The FAIR principles—findable, accessible, interoperable, and reusable—provide an important foundation for this work (Wilkinson et al., 2016), but FAIR data are not automatically meaningful data. Provenance, semantic alignment, temporal context, and fitness for a defined use determine whether shared data can support valid inference.

Longitudinal research resources have already demonstrated the value of coordinated data sharing. The Alzheimer’s Disease Neuroimaging Initiative has helped advance biomarker validation, disease staging, and reproducibility across the field (Weiner et al., 2025). Future data ecosystems should extend this model by aligning microbiome profiles, digital biomarkers, patient- and caregiver-reported outcomes, and environmental exposures around longitudinal person-level patterns rather than merely adding new modalities.

At the same time, international collaboration must protect privacy and respect local governance. Federated learning offers one promising pathway because it enables model training across institutions without moving patient-level data out of local repositories. This approach supports privacy-preserving collaboration while reducing barriers created by differing legal and regulatory frameworks (Mir et al., 2026). Federation preserves data locality; it does not by itself harmonize meaning, correct bias, or establish validity. For Alzheimer’s research, this may be especially useful because no single institution or country can fully capture the disease’s biological and clinical complexity.

## 5 Boundary conditions for a systems perspective

A systems perspective creates its own risk: complexity can become an excuse for conceptual vagueness. The framework should not imply that every pathway, modality, or data source is equally informative, nor that integration itself establishes causality or clinical utility. Models should therefore specify the biological object, temporal horizon, context of use, and dimensions simplified for analysis, and they should be judged by external validation and whether they improve decisions. This discipline keeps systems thinking falsifiable and prevents data abundance from being mistaken for understanding.

## 6 Integrating cure and care

A persistent limitation of Alzheimer’s disease research has been the uneven integration of disease-focused discovery and therapeutic development with the translation, dissemination, implementation, and scaling of evidence-based approaches to care and caregiving. Despite major advances in understanding disease mechanisms and developing interventions, substantial gaps remain between research evidence and routine practice, particularly in the implementation and sustainability of effective care models. Useful references for this perspective are (Gregory et al., 2022; Lourida et al., 2017; Gitlin et al., 2015; Boustani et al., 2021; Draper et al., 2009; National Institute on Aging, 2020; National Institute on Aging, 2021; National Alzheimer’s Project Act, 2011).

Molecular investigations have understandably concentrated on identifying disease mechanisms, biomarkers, and therapeutic targets, while care has often been viewed primarily as the responsibility of clinical practice and health systems. As precision medicine advances, however, this separation is becoming scientifically counterproductive. Earlier diagnosis, risk prediction, and individualized therapies have value only insofar as they change decisions and preserve performance; conversely, routine-care data on function, behavior, symptoms, and caregiver experience can sharpen disease models and therapeutic development. Cure and care are therefore different points in the same evidence cycle, not parallel domains.

Care should be recognized as a core objective and outcome of precision medicine rather than a secondary consequence of successful treatment. Advances in biomarkers, neuroimaging, and molecular therapeutics are essential, but their ultimate value lies in whether they preserve functional independence, improve quality of life, reduce caregiver burden, support communication and shared decision-making, and enable people living with Alzheimer’s disease to remain engaged in their communities for as long as possible. Future clinical trials and translational research should therefore evaluate patterns of cognitive, behavioral, and functional performance, quality of life, and caregiver burden alongside molecular and imaging endpoints, with attention to heterogeneity in response rather than mean effects alone.

Digital technologies illustrate how cure and care can be advanced together. Remote monitoring, speech analysis, wearable sensors, and AI-enabled clinical decision support can simultaneously generate high-quality research data, detect subtle changes in disease progression, personalize interventions, and provide timely information for caregivers and healthcare professionals. These technologies therefore serve both scientific discovery and clinical care, reinforcing rather than separating the two missions. A signal is not clinically useful merely because it can be detected; its value depends on a defined response, an accountable user, and demonstrable benefit. Successful implementation also depends on transparent AI, responsible data stewardship, and close collaboration among scientists, clinicians, patients, and caregivers.

Ultimately, Alzheimer’s disease should be addressed within a continuously learning health ecosystem in which discoveries made through research inform clinical practice, while data generated during routine care contribute to new scientific knowledge. Integrating biological, clinical, and patient-reported information in this way creates opportunities to refine predictive models, evaluate interventions in real-world settings, and improve both disease biology and lived experience. Such learning is not automatic: it requires prespecified feedback loops, provenance, external validation, and governance for how new evidence changes models and care pathways.

## 7 A practical agenda: from integration to action

To accelerate meaningful progress in Alzheimer’s disease, we propose three operational priorities.

First, define the system before building the model. Investigators should state the biological and clinical object of inference, the relevant scales and time horizons, and what their data representation preserves or simplifies. Multimodal research should then integrate molecular biology, neuroimaging, microbiology, vascular biology, clinical phenotyping, digital biomarkers, and patient-reported outcomes around a longitudinal question rather than treating modality count as a proxy for insight (Kakkar et al., 2025; Oso et al., 2026).

Second, build interoperable, federated evidence infrastructure. Progress in AI-driven precision medicine will require international collaboration supported by FAIR data principles, interoperable standards, common ontologies, evidence provenance, and privacy-preserving approaches such as federated learning (Wilkinson et al., 2016; Mir et al., 2026). Research institutions, funders, journals, and consortia should jointly support external validation, transparent governance, and equitable participation while respecting patient privacy and local control (Khachaturian et al., 2024).

Third, link every signal to a decision and every decision to a patient-centered outcome. Biomarkers, predictions, and digital measures should have a defined context of use, an accountable user, and an explicit action pathway. Their success should be judged by whether they improve patterns of functional independence, quality of life, caregiver wellbeing, equitable access, and health-system performance, not only by whether they predict molecular or cognitive endpoints (Livingston et al., 2024; Alzheimer’s Association, 2024).

## 8 A useful five-question discipline

Ultimately, we could encourage microbiology researchers to ask five questions at the end of every major research project:

1. What did we discover? Scientific contribution.
2. Why does it matter? Clinical/patient relevance.
3. Who needs to use it? User/stakeholder identification.
4. What would have to change for it to be used? Implementation requirements.
5. How will we know that it actually improved people’s lives? Real-world outcome.

That last question is particularly important.

Collaborative studies suggest that dementia research needs to overcome a “research-versus-adoption” disconnect by considering implementation across the translational continuum (Boccardi et al., 2022).

Achieving these priorities requires an operational shift toward a continuously learning health ecosystem in which molecular scientists, neuroscientists, clinicians, data scientists, patients, caregivers, and health systems work as partners. Research discoveries should inform clinical practice, while real-world performance and patient outcomes refine scientific understanding. The field will advance most effectively when cure and care are governed as a single evidence cycle with explicit feedback, validation, and accountability.

## 9 Conclusion

Alzheimer’s disease challenges traditional approaches to biomedical research because its causes, progression, and clinical manifestations emerge from the interaction of multiple biological, environmental, and social factors. No single technology or discipline will be sufficient to address this complexity. The field’s central challenge is architectural: to preserve meaningful relationships across biology, time, context, and decisions while converting knowledge into action. Progress will depend on integrating systems biology, artificial intelligence, responsible data sharing, and clinical expertise within collaborative environments that continuously generate, test, and apply new knowledge.

The next-generation of precision medicine should therefore be defined not only by its ability to identify biomarkers or develop disease-modifying therapies, but also by its capacity to change patterns that matter to people living with Alzheimer’s disease and those who care for them. Cognitive, behavioral, and functional performance, quality of life, caregiver wellbeing, and equitable access to innovation should be treated as essential measures of scientific success alongside molecular and imaging outcomes.

Advancing this vision will require sustained collaboration among molecular scientists, microbiologists, neuroscientists, clinicians, data scientists, health systems, patients, and caregivers. By integrating cure and care within a continuously learning health ecosystem, the Alzheimer’s research community can accelerate discovery while ensuring that scientific innovation delivers meaningful and lasting benefits for individuals, families, and society. The test of precision medicine is not only how precisely it classifies disease, but how reliably it changes the trajectory of a person’s life.

## Data Availability

The original contributions presented in the study are included in the article/supplementary material, further inquiries can be directed to the corresponding author.
